# Medicine

**Published:** 1905-09

**Authors:** Newman Neild


### Progress of the Medical Sciences.

### MEDICINE.

The existence of a physiological **albuminuria**[1] has now become a generally accepted fact, since it has been clearly demonstrated that the urine of all healthy persons contains albumin, although this may be present only in the most minute quantities. For instance, that deposit in urine which was

[1] *Deutsche med. Wchnschr.*, 1904, xxx. 50; Abstract in Schmidt's *Jahrbücher*, 1905.

formerly considered to be "mucus," or a "nucleo-albumin," has now been shown to be a combination of serum-albumin with certain substances which are able to precipitate albumin. Accurate analyses of urines have also demonstrated the presence of albumin, as such, in healthy individuals. This condition has been variously termed "simple," "idiopathic," "functional," or "essential" albuminuria. It is very important that a sharp distinction should be made between a merely abnormal albuminuria and an abnormal albuminuria which is caused by definite pathological conditions, a disease of the kidneys, and we must recognise that certain symptoms may be due to unusual conditions that are within the limits of health; that is, circumstances which are within the usual physiological limits. A comparison may be made on all fours with glycosuria. Just as sugar appears in the urine under certain unusual physiological conditions, so does albumin appear in the urine under certain conditions—unusual, but still within the limits of health. Thus, vigorous use of the muscles of the legs, taking and digesting a heavy meal, menstruation, a cold bath, great mental excitement, brain-fag, and similar causes may all result in the appearance of more or less albumin in the urine, according to the constitution and peculiarities of the individual. And yet these causes need not be considered pathogenic in such cases. Albuminuria under such conditions does, indeed, become pathological if it does not appear under extraordinary conditions only and does not disappear with them when they have been removed; that is, when albumin still continues to appear in the urine under normal conditions and in connection with the necessary strain of ordinary life.

As, however, physiological and pathological states may undoubtedly pass from one into the other, the consideration of this question must be confined solely to this one point of view. Thus, that form of albuminuria which is called "cyclic" or "orthostatic," and is caused by the change of position from the horizontal to the vertical, or by quiet walking, must be considered as being due to some slight irritation or inflammatory condition of the kidneys, because standing, sitting, and walking are all more or less necessary and perfectly normal things for us to do in our every-day existence. This cyclic albuminuria may certainly pass away and leave healthily functionating kidneys, but it may, on the other hand, proceed to a chronic diffuse nephritis.

In order to explain these physiological and pathological forms of intermittent albuminuria and cylindruria, it is generally accepted that there must be some peculiarity, either congenital or acquired, of the membrane surrounding the glomerulus. Senator points out that the theory of Leube, that it is all a question of an hereditary abnormality in the permeability of the glomerular membrane, is totally insufficient to explain these cases, and that we must also take into consideration the vascular system with its variations in the velocity of the blood-stream and the blood-pressure, and, moreover, its extraordinary capacity to adapt itself to the ever-varying conditions of the particular organs or of the body in general.

Thus with the change from the horizontal to the vertical posture, and on standing up, there is a general slowing down of the blood-stream in the whole of the area drained by the inferior vena cava, and this is especially noticeable in the circulation through the kidneys. During this period of venous stasis albumin will appear in the urine of those who are constitutionally predisposed thereto, but it will always cease if care is taken to increase the velocity of the blood-stream by enlarging the arteries or increasing the force or rapidity of the heart beats—by muscular exercise for instance, or by taking a hot bath, and various other measures.

Nervous irritation and brain-fag also have a great influence upon the state of the renal circulation, and without any question cold baths have a profound influence upon the circulation of the blood in the kidneys; but we are at present entirely ignorant of the precise way in which these assist in the causation of albuminuria, but there is small doubt that there are certain morphological changes in the composition of the blood which express themselves in a very mild form of hæmoglobinuria. The various changes in the velocity of the blood-stream and in the blood-pressure which take place in the kidneys during digestion are as yet but little understood, and the experiments in this direction have been few, no particular attention having been paid to this point by Pavlow or those who have followed in his methods of research upon the digestive organs. It is obvious, however, that the increase in the quantity of blood that is determined to the area drained by the portal vein, and the increased pressure in the abdomen owing to the pressure of the food, must to a certain extent increase the pressure upon the renal veins, and thereby cause a damming back of the blood in the renal circulation. Nor, in conclusion, must it be forgotten that in adults recent observations have clearly shown that there may be an abnormality of the intestine, or an idiosyncrasy, that allows certain albumins foreign to the body to pass through the intestinal mucous membrane unchanged and, entering the general circulation as foreign albumins, to be excreted as such in the urine. The examination of the urine has shown that the foreign albumin, such as that taken in the milk of various animals, is first excreted, and then, in consequence of this albumin irritating the kidney as it is excreted, ordinary albuminuria is set up. Senator asks if it is not possible that in certain individuals the intestines may similarly allow some substance or substances to pass into the circulation, thereby causing a vaso-motor disturbance of the kidneys, just as is the case with urticaria due to some food that has been taken, and hence lead to an albuminuria.

Joachim[1] has recently published some researches on the varieties of albumin present in various pathological fluids of the body. The recognition and isolation of the various kinds of albumin have not only a theoretical value, but may lead to important results clinically, as aids to diagnosis and prognosis. After explaining his methods of analysis, he enters very fully into his results, the most important of which are the following facts:—By means of a faint acidulation with acetic acid he has separated from transudates and exudates, and often from albuminous urines also, an albumin body which is identical with paraeuglobulin, a substance that can be precipitated by dilute acetic acid without boiling. This body is found in much larger quantity in exudates than in transudates. When a very strong acidulation is made it is possible to precipitate from exudates and certain urines—even from urines free from any marked quantity of serum-albumin or serum-globulin—an albumin body which Joachim considers to be a nucleo-albumin. In the pleural cavity transudates and exudates are often found to be present at the same time. The transudates are more constant in their albumin content than the exudates, and the relative quantity of euglobulin is lower in fluid from a hydrothorax than in fluid from a pleuritic effusion. In the peritoneal cavity the greatest possible quantity of albumin and the highest proportion of globulin are reached in cirrhosis of the liver. In cancer the case is exactly reversed. When the variety of forms in which tuberculous peritonitis may occur is taken into consideration, it is not surprising to learn that the results of the proteid analysis in this disease are subject to great variation. The high specific gravity and the large percentage of proteids are a proof of the inflammatory nature of the disease. In his researches on human blood serum he confirms much that is already well known. In placental blood serum and blood serum from the umbilical cord the relative proportions of albumin and globulin are about equal, but Joachim finds considerable differences in the proportion of the euglobulin to the total globulins: thus in the umbilical cord euglobulin was 56.4 per cent., whereas in the placenta it was found to be 38.3 per cent. He also made the interesting observation that when a horse had been immunised with diphtheria antitoxin there was a very marked increase in the globulin at the expense of the albumin, and, further, that there was an increase in the euglobulin. In urine, since the discovery of these proteid bodies in the blood, he has invariably been able to demonstrate albumin and pseudo-globulin in the urine of nephritic patients, the albumin usually in larger quantity than the globulin. He was unable to discover more than the faintest traces of euglobulin in urines loaded with albumin, and in most cases none whatever was found. In amyloid degeneration of the kidneys the total amount of globulin was extraordinarily increased in proportion to the

[1] *Arch. f. Physiol.*, 1903, xcii., 558; Abstract in Schmidt's *Jahrb.*, 1905.

albumin, this increase being due not to the pseudo-globulin but to the euglobulin.

There are numerous researches being carried out at the present time, endeavouring to separate and identify the various globulins of the blood serum. Otto Porges and K. Spiro[1] have found that by fractional precipitation with ammonium sulphate three groups can be separated. The first begins to fall at 28 per cent., the next at 33 per cent., and the last at 40 per cent., the percentage at which precipitation begins being in each case sharply defined; and, although no definite chemical differences have as yet been found, there are some indications that this may be accomplished.

Vignier[2] has described an epidemic of **benign infectious jaundice.** A company of soldiers mounted a battery on a piece of ground where there had formerly been a latrine, and amongst these men there occurred an epidemic of jaundice. He maintains that the probable cause of the epidemic was the bacillus coli communis, which must have retained its virulence for many years. The contagious nature of the disease was shown by the fact that five soldiers who had not assisted in working the battery, but who came in contact with them afterwards, contracted the disease.

The onset of the disease was not acute, for the duration of the various prodromal symptoms extended over three to fifteen days. The actual symptoms of the disease, however, lasted only three or four days, but convalescence was extraordinarily protracted. The chief symptoms were rapid loss of strength, loss of appetite, headache, jaundice, abdominal pain, constipation, colourless or bile-stained fæces, and diminished urine. A slow pulse and some fever completed the picture of the disease, and these symptoms were present in all of the cases observed. Towards the end of the epidemic some of the patients began with diarrhœa, but this was followed by a more prolonged constipation. The colour of the stools varied according to the jaundice, and ranged between absolute colourlessness and an almost black colour. There was no meteorism, the liver was not tender, and, if anything, was diminished in size rather than enlarged. The pain was felt chiefly in the epigastrium, and was very severe for three or four days, the tenderness to palpation remaining for a longer period.

Vignier in a later paper discusses the origin of the pain. He does not consider that it can be attributed either to the stomach or the liver, but says that it is due to a swelling of the pancreas, because that organ lay immediately beneath the

[1] *Beitr. z. chem. Physiol. u. Patholog.*, 1903, iii. 4 and 6; Abstract in Schmidt's *Jahrb.*, 1905.

[2] *Arch. de Med. et de Pharm. mil.*, xliii. 6, p. 429; Abstract in Schmidt's *Jahrb.*, 1905.

area of tenderness. The presence of jaundice might have been urged to account for the bradycardia, but he prefers to attribute this also to the swelling of the pancreas, the enlargement of the pancreas being supposed to irritate the solar plexus and thereby slow the pulse; at any rate, such a result has been shown to occur experimentally—the so-called "symptome d'irradiation solaire" of Lacquel-Lavastine.

The nomenclature of this disease is due to a comparison of it with Weil's disease, often termed **infectious febrile jaundice,** and of a most malignant character. Hitherto Weil's disease has not been observed at an earlier age than 8 years, and 90 per cent. of the cases have occurred in men between the ages of 20 and 30, but Brüning[1] has now placed on record an unique case occurring in a child under 12 months. The case was an infant 4 months old, which was diagnosed during life as having Weil's disease, and the diagnosis was verified by a *post-mortem* examination.

Pathologically the case was primarily a fatty degeneration and fatty infiltration of the liver, and a well-marked diffuse purulent inflammation of the parenchyma of the kidneys. The lymphatic follicles of the intestines were swollen, and there were an infiltration and necrosis of the mucous membrane surrounding hemorrhages. Cultures were made from the blood both during life and at the *post-mortem* examination with resulting cultures of bacillus proteus fluorescens, the organism which has already been established by Jaeger as the cause of Weil's disease in adults. When this organism is injected subcutaneously and intraperitoneally in animals, there seems to be no formation of toxin by the bacillus, but if, on the other hand, animals are fed with this organism an acute enteritis resulted, showing that Weil's disease is probably caused by an infection of the alimentary tract. NEWMAN NEILD.

